# Focal Liver Lesion MRI Feature Identification Using Efficientnet and MONAI: A Feasibility Study

**DOI:** 10.3390/cells11091558

**Published:** 2022-05-05

**Authors:** Róbert Stollmayer, Bettina Katalin Budai, Aladár Rónaszéki, Zita Zsombor, Ildikó Kalina, Erika Hartmann, Gábor Tóth, Péter Szoldán, Viktor Bérczi, Pál Maurovich-Horvat, Pál Novák Kaposi

**Affiliations:** 1 Medical Imaging Centre, Department of Radiology, Faculty of Medicine, Semmelweis University, 1083 Budapest, Hungary; budai.bettina@med.semmelweis-univ.hu (B.K.B.); ronaszeki.md@gmail.com (A.R.); zita.zsombor7@gmail.com (Z.Z.); ilkalina@t-online.hu (I.K.); hartmann.erika@gmail.com (E.H.); toth.gabor.dr@gmail.com (G.T.); berczi@hotmail.com (V.B.); maurovich-horvat.pal@med.semmelweis-univ.hu (P.M.-H.); kaposi.pal@med.semmelweis-univ.hu (P.N.K.); 2 MedInnoScan Research and Development Ltd., 1112 Budapest, Hungary; peter.szoldan@medinnoscan.com

**Keywords:** focal liver lesion, deep learning, radiological feature, hepatocellular carcinoma, liver metastasis, gadoxetate disodium, abdominal MRI, multidimensional imaging

## Abstract

Liver tumors constitute a major part of the global disease burden, often making regular imaging follow-up necessary. Recently, deep learning (DL) has increasingly been applied in this research area. How these methods could facilitate report writing is still a question, which our study aims to address by assessing multiple DL methods using the Medical Open Network for Artificial Intelligence (MONAI) framework, which may provide clinicians with preliminary information about a given liver lesion. For this purpose, we collected 2274 three-dimensional images of lesions, which we cropped from gadoxetate disodium enhanced T1w, native T1w, and T2w magnetic resonance imaging (MRI) scans. After we performed training and validation using 202 and 65 lesions, we selected the best performing model to predict features of lesions from our in-house test dataset containing 112 lesions. The model (EfficientNetB0) predicted 10 features in the test set with an average area under the receiver operating characteristic curve (standard deviation), sensitivity, specificity, negative predictive value, positive predictive value of 0.84 (0.1), 0.78 (0.14), 0.86 (0.08), 0.89 (0.08) and 0.71 (0.17), respectively. These results suggest that AI methods may assist less experienced residents or radiologists in liver MRI reporting of focal liver lesions.

## 1. Introduction

The number of focal liver lesions (FLLs) detected with imaging studies is steadily growing worldwide, and accurate diagnosis is crucial not only for avoiding treatment delay but also for sparing patients and health care providers from unnecessary procedures and lowering costs. Today, magnetic resonance imaging (MRI) offers the most comprehensive non-invasive characterization of FLLs, and, contrary to computer tomography (CT), it does not expose patients to radiation. MRI imaging, performed using a selection of pulse sequences and often with intravenous contrast agents, provides excellent soft-tissue resolution and results in a highly accurate diagnosis, with a sensitivity of 94% and a specificity of 82–89% [1].

Hepatocyte-specific contrast agents (HSCs) have been proved highly useful in lesion detection and differentiation between benign and malignant foci, and they have been increasingly used for imaging of FLLs. These contrast agents, including gadoxetate disodium and gadobenate dimeglumine, are taken up by functioning hepatocytes and are excreted into the bile [2]. Thus, HSCs can be used to differentiate between liver lesions consisting of normal hepatocytes and those containing poorly differentiated hepatocytes or cells with non-hepatocytic origin. It is also easier to detect small lesions because there is a strong contrast between the enhancing parenchyma and foci without normal hepatocytes in the hepatobiliary phase (HBP) [2].

Some common FLLs, such as hepatocellular carcinoma (HCC) and liver metastasis (MET), constitute a significant diagnostic challenge, as these patients can only be cured with surgical resection or image-guided ablation; thus, the significance of early detection is paramount. METs are the most common malignancies of the liver. It has been reported that up to 70% of all patients with colorectal cancer would develop METs at some point in their lifetime [3]. HSC-enhanced MRI has been shown to have the highest sensitivity (73.3%) for METs smaller than 10 mm in diameter among all imaging modalities, which also translates to a significantly better survival rate of patients imaged with MRI (70.8%) compared to those imaged with CT (48.1%) [4]. HCC is the fifth most common solid malignancy worldwide, and the mortality rate from HCC is predicted to rise in the coming decades [5]. HCC typically develops in the background of decades of chronic liver disease (CLD). MRI findings of an arterial-enhancing mass with subsequent washout and enhancing capsule on delayed interstitial phase images are diagnostic for HCC [6]. Focal nodular hyperplasia (FNH) is the second most common benign solid FLL after hemangioma. FNH is a common incidental finding in imaging studies, and it is a frequent source of differential diagnostic dilemmas of malignant lesions. A definitive diagnosis of FNH can be established in patients who do not have CLD when typical features such as arterial phase and HBP hyperenhancement and a central scar are detected with HSC MRI [7]. Standardized data collection and reporting systems have also been developed, such as the Liver Imaging Reporting and Data System (LI-RADS), to improve CT and MRI diagnosis by reducing variability in the interpretation of imaging studies [8]. However, due to the complex nature of these systems, their integration into the clinical workflow can be cumbersome.

Artificial intelligence (AI) techniques have been introduced in growing numbers to facilitate lesion detection and classification, assess the patients’ prognosis, or identify risk factors of FLLs based on imaging studies [9]. Some of these studies extracted large numbers of image features from dynamic contrast-enhanced MRI to build mathematical models for the automatic classification of FLLs [10]. Deep learning models (DLMs) are state-of-the-art image processing algorithms predominantly based on convolutional neural networks (CNN). DLMs have been tested for analysis of all known imaging modalities and achieved excellent results in image-based detection and classification of various diseases [11]. A handful of studies have also applied DLMs to classify FLLs in MRI images and demonstrated that the performance of the DLMs is excellent and comparable to the human observers’ diagnostic rate [12,13,14,15]. Among different DLM architectures, models using 3D convolutions could be efficiently trained on a relatively small number of cases for differentiating between the most common types of FLLs [14,15].

Meanwhile, current AI classification models have limited value in clinical practice as these have been trained to diagnose only a handful of liver pathologies based only on a small set of MRI images. Current DLMs cannot recognize many FLLs belonging to less common diagnoses and lesions with atypical image features or with post-treatment changes [16]. A clinically useful DLM must be able to analyze multiple image sequences to identify a comprehensive set of image features that can be used for the characterization of FLLs and the generation of a differential diagnosis [17]. For the transparency of the AI-driven classification process, it is essential to know how many of the detected image features support the diagnosis and to validate the localization of these features via network visualization techniques, such as activation and occlusion sensitivity maps. Such sophisticated models are better suited for the systematic evaluation of FLLs and can increase the efficiency and the reproducibility of the imaging diagnosis.

Previously, we have shown that DLMs using multi-sequence HSC MRI images can accurately differentiate between FNHs, HCCs, and METs [15]. In the present study, we aim to demonstrate that 3D CNNs can be applied for comprehensive evaluation and visualization of diagnostic image features in the same three types of FLLs in HSC MRI. We also investigate the consistency between the detected image features and the predicted diagnosis in each class, and the agreement between the DLM and radiologists.

## 2. Materials and Methods

### 2.1. Clinical Dataset

For our retrospective study, 99 patients were included (Table 1) who underwent abdominal MRI with gadoxetate disodium, a hepatocyte-specific contrast agent (HSC), between 29 September 2017 and 11 August 2021, at our institution. As this is a retrospective study, the need for written patient consent for this retrospective analysis itself was waived by the Institutional Research Ethics Committee. However, all patients gave written informed consent for the MRI examination. The study was conducted in accordance with the Declaration of Helsinki and approved by the institutional review board of our university. Inclusion criteria of the study covered patients who were examined for FLLs with HSC-enhanced MRI in our institution (134 patients, 175 examinations) using the same 1.5 T MRI machine, a Philips Ingenia 1.5 T scanner (Philips Medical Systems, Eindhoven, The Netherlands), and whose liver lesions could be unequivocally diagnosed based on histology sampling or typical imaging findings as it has been recommended by international guidelines. Exclusion criteria included age under 18 years at the time of the imaging, pregnancy, incomplete or inadequate quality scans, data collection errors, examination performed on a different MRI machine, and lesions with an equivocal diagnosis. Fifteen examinations were excluded as they were performed on a different scanner, 21 studies were excluded due to incomplete or inadequate scan quality and data collection errors, while 4 studies were performed on underage patients, and 4 studies did not contain or only contained lesions with an equivocal diagnosis.

The final study cohort included 131 scans of 99 subjects diagnosed with 105 FNHs, 121 HCC, 121 METs, and 32 other lesions belonging to various groups (such as hemangiomas and adenomas).

### 2.2. Image Acquisition and Processing

All MRI scans were acquired using a Philips Ingenia 1.5 T scanner (Philips Medical Systems, Eindhoven, The Netherlands) and 5–20 mL intravenous gadoxetate disodium contrast with a dosage of 0.025 mmol/kg (Primovist ^TM^, Bayer A.G., Berlin, Germany). The scans were performed according to our institutional guidelines. For the current study the T2-weighted (T2w) spectral-attenuated inversion recovery (SPAIR), native T1-weighted 3D mDIXON water only (NAT), arterial (ART), portal venous (PVP), equilibrium phase (VEN) T1-weighted 3D mDIXON, as well as hepatobiliary phase (HBP), standardly acquired at 15–20 s (ART), 70–80 s (PVP), 2–3 min (VEN) and 20 min (HBP) after contrast administration, images of each lesion were collected from the institutional picture archiving and communications system (PACS). Both T2w and T1w scans were acquired in breath-hold. Standard 3D mDIXON and T2 SPAIR sequences were used. 3D mDIXON: 390 × 390 × 106 average image resolution, 0.942 mm × 0.942 mm average pixel spacing, 2.5 mm average spacing between slices, 4–6 mm slice thickness, 5.8 ms repetition time (TR), 1.8/4.0 ms echo time (TE), 15° flip angle and 552–616 Hz/pixel receiver bandwidth. T2 SPAIR: 398 × 398 × 100 average image resolution, 0.935 mm × 0.935 mm average pixel spacing, 2.8 mm average spacing between slices, 3–6 mm slice thickness, 1000–6742 ms TR, 100 ms TE, 90° flip angle, and 325–666 Hz/pixel receiver bandwidth.

**Figure 1:**
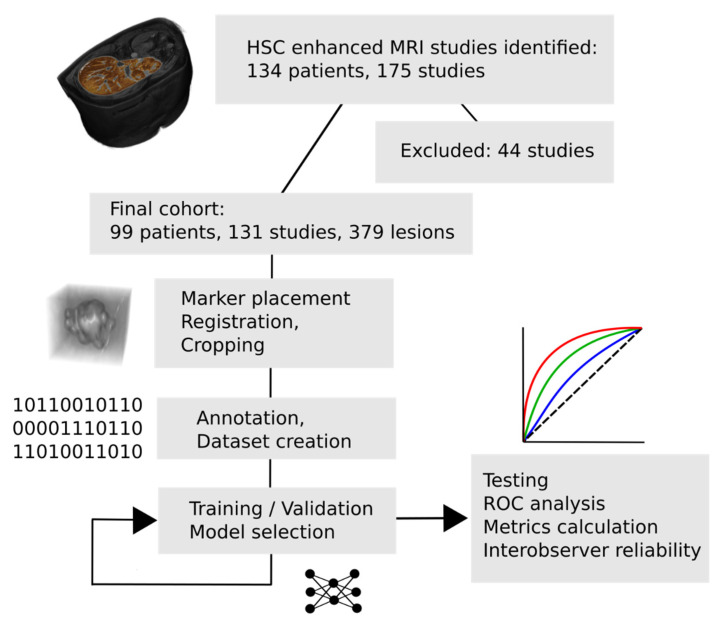
Main steps of the study. After the selection of analyzable examinations, each was annotated and preprocessed, and the marked lesions were cropped for training. The trained model was evaluated on a separate test dataset. HSC = hepatocyte-specific contrast-enhanced, MRI = magnetic resonance imaging.

Each scan was anonymized, and personal identifiers, such as patient name, birth date, social security number, and date of imaging were removed using the PyDICOM [18] (www.pydicom.github.io, accessed on 26 October 2021) Python (Python Software Foundation, Wilmington, DE, USA) module. All scans were converted to 3D NIfTI [19] image format, HBP scans were resampled with linear interpolation to isotropic, 1 × 1 × 1 mm voxel spacing (using Simple ITK [20]), and all other scans were coregistered to the corresponding HBP scan. For image registration, the ITKElastix toolbox [21] was used with the rigid default parameter map. When coregistration was insufficient, scan misalignment was manually corrected. Each scan where FLLs were reported was marked (markers were placed in the HBP or ART scan), and their diameter was manually measured by an expert radiologist with 13 years of experience in abdominal imaging. Only lesions with the largest diameter of at least 5 mm were included in the study. Lesions were cropped from each scan based on their largest diameter, to which a 2-mm (2 voxels)-wide zone was added in each direction as a safety margin for misalignments between the 6 scans. Manual correction and lesion marking were performed using 3D Slicer [22] (www.slicer.com, accessed on 26 Octorber 2021). The cropped scans, along with the dataset annotations and the trained model, are openly available through FigShare (www.figshare.com, accessed on 1 April 2022) at the following DOI: 10.6084/m9.figshare.19495013 (accessed on 31 March 2022). Figure 1 summarizes the steps of the analysis.

Cropped lesions were randomly sorted into training, validation, and test datasets for AI training and testing (Table 2) in a ratio of 53:17:30. In the MRI scans of 99 patients, the total number of focal liver lesions was 379, 202 lesions were used for training, 65 for validation, and 112 for testing. All scans and lesions belonging to the same patient were assigned to the same dataset to avoid data leakage. Each lesion was evaluated by an expert radiologist with 13 years of experience in abdominal imaging, as well as a radiology resident with 4 years of experience. Annotators had to decide whether the tumor belonged to the four provided tumor types: FNH, HCC, MET, or other; and whether or not the lesion contained the following radiological features: early (arterial phase) contrast enhancement, washout, delayed phase enhancement, peripheral enhancement, central scar, capsule, T2 hyperintensity compared to the surrounding liver tissue, iso- or hyperintensity compared to the surrounding liver tissue on venous phase, hypoenhancing core, hemorrhage/siderosis. The human observers were blinded from the final diagnosis and each others’ results. For AI training, the expert-reported labels were used as ground truth, as well as for results calculation. Table 2 contains the detailed distribution of expert annotations among datasets.

### 2.3. Deep Learning Methods

To be able to automate radiological feature generation, multiple deep learning algorithms were trained with different hyperparameter setups. All models used were implemented in the Medical Open Network for Artificial Intelligence (MONAI), a Pytorch-based open-source framework for deep learning in healthcare imaging. MONAI (monai.io, accessed on 17 December 2021) provides multiple 3D neural network implementations that are capable of performing classification tasks on medical images. Each trained convolutional neural network had 6 input channels, one for each 32 × 32 × 32 resolution cubic input MRI scan showing the liver lesion. We trained DenseNet121 [23], DenseNet169, DenseNet201, DenseNet264, EfficientNetB0 [24], EfficientNetB1, EfficientNetB2, EfficientNetB3, EfficientNetB4, EfficientNetB5, EfficientNetB6, EfficientNetB7 models with various batch sizes, dropout rates and weight decay, among other hyperparameters. All models were trained for at least 300 epochs. Each model was modified so that its last classifier layer would be a sigmoid layer to be able to perform multi-label classification. Each model has 10 probability outputs (numeric values ranging from 0 to 1), one for each radiological feature that it is trained to predict. By replacing (occluding) a part of the input volume with the mean voxel intensity of the image, the model prediction for each radiological feature changes. If important parts of the image are occluded, the prediction probability decreases, which can be visualized for each input channel and output feature. More negative values indicate higher importance in the decision process. This visualization technique will be referred to as ‘occlusion sensitivity map’ in the Section 3. These maps can be useful for the interpretation of network predictions, highlighting the areas that played a more important role in the prediction of a feature [25]. Accordingly, binary cross-entropy (BCE) loss was calculated and used for model weight adjustment during training. 

DenseNets were trained with dropout probabilities of 0, 0.25, 0.5, and 0.75. Each model was trained using an Adam optimizer and a learning rate of 0.0001. All models were trained from scratch, without pretraining. To prevent overfitting and improve the generalizability of the model, different data augmentation methods (such as rotation of the images) were applied during training. Images were then resized to a 32 × 32 × 32 input shape. Image intensities were normalized and scaled between −1 and 1. The best-performing model was defined as the one achieving the highest mean area under the receiver operating characteristic curve (AUC) for the 10 predicted features on the validation dataset. Validation dataset performance was evaluated every 20 epochs. The diagnostic performance of the final model was evaluated on an independent test dataset from the same database (Table 2).

The code of the classification algorithm is, in part, based on MONAI (version 0.8.0) and other open-source projects, and is available at https://github.com/stollmayer/feature_identifier (accessed on 31 March 2022). To replicate the test results, please visit the GitHub repository link. Experiments were performed using an AMD Ryzen™ 9 5900X processor (Advanced Micro Devices Inc., Santa Clara, CA, USA) and a GeForce RTX™ 3060 12 GB graphical processing unit (Nvidia Corporation, Santa Clara, CA, USA), along with 64 gigabytes of random access memory on Ubuntu 20.04 (Canonical Ltd., London, UK). Analysis was performed using custom-written software in Python 3.8.8 and R version 3.6.3 (The R Foundation, Indianapolis, IN, USA). For a detailed description of software packages, please refer to the project’s GitHub repository.

### 2.4. Statistical Evaluation

For statistical evaluation of the test dataset, receiver operating characteristic curve (ROC) analysis was performed, AUC values were calculated, and cut-off values were set for each feature separately based on Youden’s index. At the given thresholds, sensitivity, specificity, positive predictive value (PPV), negative predictive value (NPV), and f1 score were calculated for each feature. Reported measures are calculated in comparison to the expert radiologist’s opinion (ground truth). Power calculations were carried out according to Obuchowski’s method using the ‘pROC’ [26,27] R package. Inter-rater reliability analysis was performed by calculating Cohen’s Kappa between expert opinion, annotations by a radiology resident, and the machine learning model.

## 3. Results

**Figure 2:**
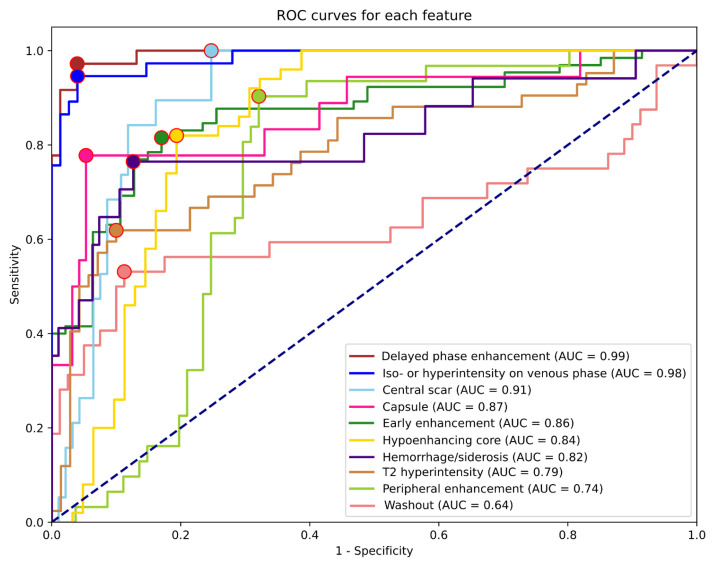
Receiver operating characteristic (ROC) curves for each feature are based on the test dataset predictions. Dots indicate the cut-off points used to calculate metrics for the specific feature.

After training each model with multiple hyperparameter setups, the highest validation mean AUC (0.9147) was achieved by the EfficientNetB0 model after 480 epochs. In this setting, the network was trained with a batch size of 32. We provide the training results of the other model architectures as well in decreasing order, based on validation mean AUC: EfficientNetB6 (0.9033), EfficientNetB2 (0.9033), EfficientNetB3 (0.902), EfficientNetB4 (0.8988), EfficientNetB1 (0.8922), EfficientNetB5 (0.8922), DenseNet121 (0.8807), DenseNet169 (0.8792), DenseNet201 (0.8733), DenseNet264 (0.8682), EfficientNetB7 (0.856). The final EfficientNetB0 model could identify most features with excellent metrics when tested on the independent test dataset. Table 3 summarizes the results for each feature, including all lesion types. The highest AUCs were reached for the detection of delayed phase enhancement (0.99) and iso- or hyperintensity on the venous phase (0.98). These features were only rarely detected as false positives or remained undetected. The least predictable features based on AUC were T2 hyperintensity (0.79), peripheral enhancement (0.74), and washout (0.64). ROC curves and corresponding AUC values are shown in Figure 2.

The highest and lowest PPVs were reached for delayed phase enhancement (0.92) and central scar (0.44) detection, while the best and worst sensitivities were for central scar (0.95), delayed phase enhancement (0.94), and iso- or hyperintensity on venous phase (0.92) vs. T2 hyperintensity (0.60) and washout (0.50). NPVs and specificities were higher on average (0.89, 0.86) than PPVs and sensitivities (0.71, 0,78). Apart from early enhancement (0.75) and T2 hyperintensity (0.79), all other NPVs were above 0.8. The feature with the lowest specificity was peripheral enhancement (0.68), while the most specific was delayed phase enhancement (0.96). As shown in Table 4, almost all feature AUCs were calculated with power reaching 0.98; therefore, the number of samples is more than sufficient to support these results.

**Figure 3:**
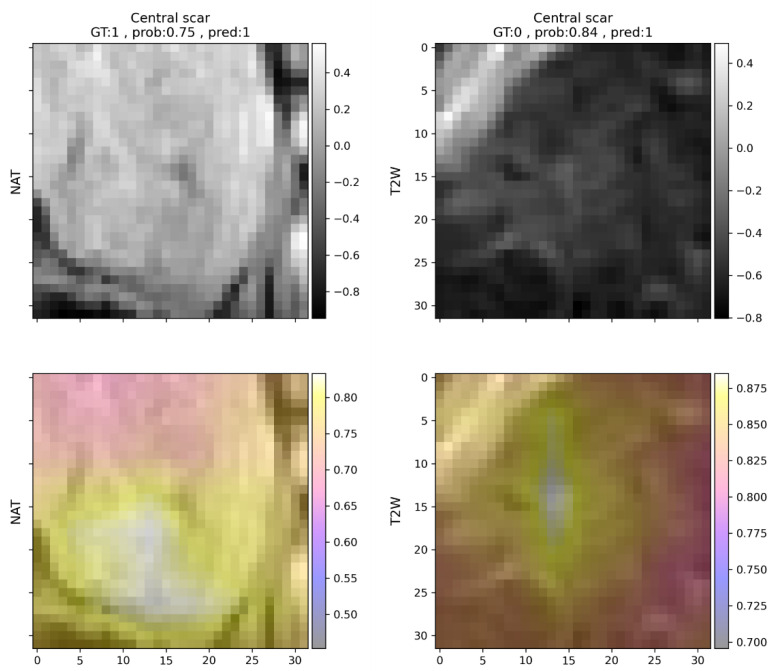
Two examples (in each column) of central scar predictions in focal nodular hyperplasias. Left: correct prediction, right: incorrect prediction. Upper row: native T1-weighted (**left**) and T2-weighted (**right**) images. Rescaled voxel intensities are indicated on the *y*-axis. Lower row: occlusion sensitivity maps indicating the contribution of each voxel to the prediction. In the case of the T2-weighted image, the area representing the central scar presumably increases the probability of the identification of this feature. In the case of the native T1-weighted image, the areas near the central scar led to the highest increase in the prediction probability. GT = ground truth, prob = probability, pred = prediction, NAT = native T1-weighted image, T2W = T2-weighted image.

To be able to explore the differences in predictions between the different lesion types, results are reported for FNHs, HCCs, and METs separately as well (Table 4, Table 5 and Table 6). Since not all features are present in all lesion types, not all metrics can be calculated for all features in each case. To simplify this problem, feature predictions are ordered according to their respective f1 scores. To provide more details on false detections, non-abundant features are also listed for each lesion type. Features present in FNHs were generally well recognizable by the model. Features related to contrast enhancement that are representative of FNHs, such as early or delayed phase enhancement, had f1 scores above 0.95, while non-present features were rarely detected. Central scars were common false positive detections, but mostly if the lesion was FNH (Figure 3). If the lesion analyzed was HCC (Table 5) or MET (Table 6), the model almost never predicted the presence of a central scar.

Among all lesion types, HCC feature prediction yielded the least desirable results. As reported in Table 5, diagnostically important features, namely washout and early enhancement, were undetected in half and nearly half of all cases. Features that are present in both HCCs and METs, such as peripheral enhancement (MET), were common false positive findings in the HCC group, but not in the MET group. Capsule was less difficult to detect, but peripheral enhancement was falsely detected in half of the analyzed cases, possibly due to the similarity between the two. Although hemorrhage was reported only in HCCs by the expert annotator, the algorithm predicted it in four cases in FNHs as well, and two cases in METs. Hemorrhage in HCCs remained undetected in one-third of cases, similar to hypoenhancing core (Table 5). Features related to contrast enhancement were detected less accurately in HCCs. Hypoenhancing core was missed in five cases and falsely detected in nine cases. The presence of other similar features such as early enhancement or hemorrhage might make the detection of a hypoenhancing core more difficult.

**Figure 4:**
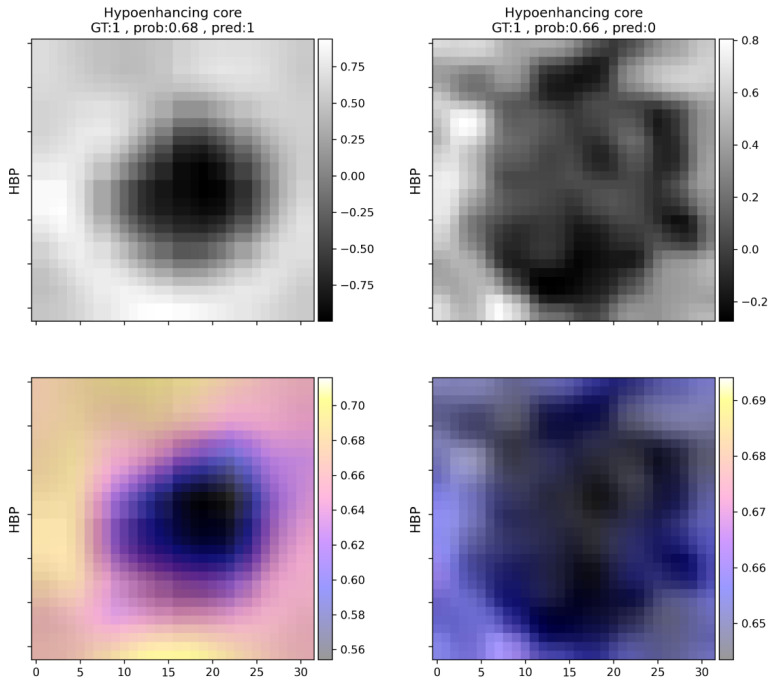
Examples of hypoenhancing core predictions in liver metastasis (**left**) and hepatocellular carcinoma (**right**). **Left**: correct prediction, **right**: incorrect prediction. Upper row: processed hepatocyte-specific contrast-enhanced scans. Rescaled voxel intensities are indicated on the *y*-axis. Lower row: occlusion sensitivity maps indicating the contribution of each voxel to the prediction. These maps indicate the prediction probability of the model for the hypoenhancing core feature, while the corresponding part of the image is replaced by the mean intensity value of the image. In the shown cases the image area that represents the hypoenhancing core is replaced by higher values (which makes the hypoenhancing core disappear), thus decreasing the probability of the identification of this feature. GT = ground truth, prob = probability, pred = prediction, HBP = hepatocyte-specific contrast-enhanced image.

The most common mistake in the case of METs was the underdiagnosis of T2 hyperintensity (eight cases), which was most commonly marked in this group (Table 6). Features mostly present in FNHs were almost perfectly predicted (Table 4), while washout and early enhancement were the most common falsely detected features. Both peripheral enhancement and hypoenhancing core were identified with an f1 score above 0.9. For an example of hypoenhancing core prediction, see Figure 4.

Features reported in other lesion types were variably predictable (Table 7). Peripheral enhancement might be confused with nodular enhancement, exhibited by hemangiomas, which was not explicitly analyzed, as only a low number of cases were available. Hypoenhancing core represents a similar case, as both cysts and hemangiomas may mislead the model predictions due to their enhancement characteristics.

To assess the agreement between the model, a radiology resident, and an expert radiologist with substantial experience in abdominal radiology, Cohen’s Kappas were calculated for each feature in each combination (Table 8). The mean score was 0.60 for the agreement between the predictive model and the expert, similar to novice opinion compared to model predictions, indicating moderate reliability. In the case of delayed phase enhancement and venous phase iso- or hyperintensity, the agreement was almost perfect (>0.8). Even the worst feature predictions (central scar, peripheral enhancement, capsule) showed moderate agreement (>0.4) with the expert opinion. Features that were less accurately predicted by the network were also subject to disagreement between the two human observers. Central scar, for example, was more frequently identified by both the model and the radiology resident, while only moderate agreement was observable in the case of washout in all three comparisons.

Generally, the described model predictions are reliable, and they could provide descriptions of radiological features present in FLLs, putting more weight on the exclusion of a feature and allowing false positive predictions depending on the type of lesion and features present. It must also be mentioned that the listed mistakes may partly be due to human uncertainty or the lack of consensus among experts on the definition of a given radiological feature, not to mention various imaging artifacts and image processing errors that may make proper predictions more difficult.

## 4. Discussion

The current paper explores our findings on how DLMs may perform on a small, single-institutional dataset concerning a complex reporting task. HSC MRI-based approaches are not novel in abdominal radiomics. Several research groups have reported excellent results on the automatic, DLM-based classification of various types of FLLs, but these put more emphasis on predicting lesion class and less emphasis on mimicking the human observers [12,14,15,28]. More interpretable methods have been described in radiology in general, the most obvious one being chest X-ray reporting using deep learning methods, where multiple findings have to be identified in parallel by the AI [29]. While chest X-ray interpretation is among the most advanced research areas in deep learning radiomics, other examination types and areas with less frequently performed studies and much more complex reporting tasks lack sufficient proof for the application of AI methods. Research on radiological feature descriptors is also of importance as many of the lesions are multifocal, many types may be found parallel, and histological confirmation cannot be acquired in all cases, thus, a certain diagnosis may not be possible (and necessary) for all lesions. Additionally, the described features allow a much broader extension of applications, since each may allow the user to draw different conclusions, such as whether tumor recurrence is observable (enhancement) or whether the malignant transformation of a regenerative nodule has occurred.

These are partly the reasons why the main emphasis of the current paper is on radiological feature identification. Although the classification of different FLLs based on the identified features could seem like a straightforward task, various challenges promote it to a research topic on its own. While the majority of the lesions evaluated in our study fell into three main lesion types, the liver is host to one of the largest varieties of focal pathologies; as such, it would be worth examining diagnostic algorithms built upon the present feature identifier in a more detailed manner. As such, they should be evaluated on a larger variety of pathologies. There are multiple lesion types, for example, cholangiocellular carcinoma, that are not present in the current dataset, but in future studies should be evaluated, considering their clinical importance. Apart from this, further evaluation in this direction could be carried out in multiple ways that did not fit within the scope of the current manuscript. A classifier model could be built solely for the diagnosis of FLLs, as well as by reusing the currently presented feature identifier, for example, via transfer learning. In this case, the training of the model would be guided to take into account the radiological features identifiable by human observers, apart from deep features. The diagnosis of the tumor could also be based on the probabilities of the predictions for each feature. In this case, the top-N features would be used to create an algorithmic approach for diagnosis making. Our current interpretation of the feature detector partly opposes this approach, as the predictions of the model are evaluated based on the calculated optimal cut-off values. Apart from these, there could be other ways to create a diagnostic model that integrates the feature identifier for better interpretability. Because of this, the automatic classification of focal liver lesions lies outside the scope of the current paper.

Abdominal imaging studies, such as HSC MRI, are less frequently approached in a similar manner due to the higher cost of imaging, the complexity of the task, the smaller amount of available data, and the more variable agreement on radiological feature abundance among professionals, as well as the need for more time-consuming data preparation and analyses. Most papers use some form of deep learning interpretation method, such as attention maps, to try to find explanations for classification predictions, while direct feature predictions have rarely been the focus of research. Wang et al., in their 2019 study, were among the first to use CNNs for focal liver lesion feature identification [17]. The reported model was able to correctly identify radiological features present in test lesions with 76.5% PPV and 82.9% sensitivity, which is similar to our results, though their method was built on a precious lesion classifier, from which feature predictions were derived. Our study deliberately avoided the diagnosis of lesions and focused solely on feature identification. Sheng et al. also used deep learning to predict radiological features based on gadoxetate disodium-enhanced MRI, dedicated to LI-RADS grading in an automated and semi-automatic manner. They reported AUCs of 0.941, 0.859, and 0.712 (internal testing) for arterial phase enhancement, washout, and capsule prediction. The model was also tested on an external test set, achieving AUCs of 0.792, 0.654, and 0.568, respectively [30]. Though they evaluated fewer features, similarly to our findings, arterial phase enhancement was more accurately predictable than washout and capsule, both of which are challenging for the AI to predict. The results of Wang et al. also led to a similar conclusion, as arterial phase hyperenhancement and delayed phase hyperenhancement, among others, just as according to our results, were well predictable features, while others, such as central scar and washout, were especially difficult to accurately predict [17]. Central scar and washout were also fairly difficult to identify and were quite often false positive findings; furthermore, in our experience, circle-like features such as peripheral enhancement, which might be confused with capsule by the model, were just as common false positive findings. The difficulty in the detection of these features is consistent with previous research on gadoxetate disodium, as HCC indicative features, such as capsule and washout, are less distinguishable using gadoxetate disodium than with extracellular contrast agents [31]. Delayed phase enhancement, which is related to the hepatocyte-specific nature of gadoxetate disodium, was an accordingly straightforward prediction.

In the current study various occlusion sensitivity maps are shown that attempt to visually explain the decision-making process of the neural network classifier. The maps can be helpful in explaining the decision-making process even in a very complex task and can draw attention to erroneous decision-making that may be based on, for example, image artifacts or non-task-related image areas. The modification of the padding value from the image mean intensity to specific values depending on scan type and predicted radiological feature may be a promising direction for further investigation.

As mentioned previously, features on which there might be disagreement between expert radiologists as well (e.g., central scar) are more difficult to build a model upon. In the future, it is possible that more thorough curation of training data based on the opinion of multiple experts would be necessary to optimize these methods. A promising research direction would be a more detailed examination of how each image, their quality, and the reported expert consensus could be used to construct balanced, high-quality datasets that are more representative of radiological liver lesion features. The current study has additional limitations. It was retrospectively conducted within a single institute, and only a small number of patients were included. To mitigate the consequences of these problems, further multi-institutional studies are needed. Additional methods, such as transfer learning with other, similar, multi-modal datasets may be used in addition to the previously mentioned dataset reannotation. Further data augmentation methods, such as random cropping, also have to be evaluated. Splitting the model into multiple feature predictors based on conflicting features and corresponding scans may also be examined as a potential solution for inaccurate predictions (e.g., T2 hyperenhancement and hypoenhancing core). Apart from these, the tested methodology has the potential to aid less experienced radiologists or other clinicians in understanding and interpreting HSC MRI of FLLs in an automated, controllable manner by providing predictions of radiological features in a few seconds.

## Figures and Tables

**Table 1:** Patient demographics and types of lesions analyzed in the study. Some patients were diagnosed with multiple lesion types; therefore, the number of included patients is not equal to the sum of the number of patients diagnosed with different lesion types.

	FNH	HCC	MET	Other	All Patients
Number of patients	52	23	17	16	99
Male	15	16	9	6	42
Female	37	7	8	10	57
Average age at the time of imaging	44	64	57	53	54

FNH = focal nodular hyperplasia, HCC = hepatocellular carcinoma, MET = liver metastasis.

**Table 2:** Distribution of lesions and annotated features among datasets.

Tumor Type	Train	Validation	Test	Total
FNH	53	16	36	105
HCC	62	22	37	121
MET	72	19	30	121
Other	15	8	9	32
Radiological features				
Early enhancement	99	36	65	200
Washout	41	8	32	81
Delayed phase enhancement	65	28	36	129
Peripheral enhancement	53	21	31	105
Central scar	37	11	19	67
Capsule	27	6	18	51
T2 hyperintensity	88	39	42	169
Iso- or hyperintensity on venous phase	64	28	37	129
Hypoenhancing core	110	28	50	188
Hemorrhage/Siderosis	36	17	17	70

FNH = focal nodular hyperplasia, HCC = hepatocellular carcinoma, MET = liver metastasis.

**Table 3:** Test dataset metrics and statistical power calculation.

Radiological Features	PPV	NPV	Sensitivity	Specificity	f1	AUC	Power (*p* = 0.05)
Delayed phase enhancement	0.92	0.97	0.94	0.96	0.93	0.99	1
Iso- or hyperintensity on venous phase	0.92	0.96	0.92	0.96	0.92	0.98	1
Central scar	0.44	0.99	0.95	0.75	0.60	0.91	1
Capsule	0.72	0.95	0.72	0.95	0.72	0.87	1
Early enhancement	0.87	0.75	0.80	0.83	0.83	0.86	1
Hypoenhancing core	0.77	0.83	0.80	0.81	0.78	0.84	1
Hemorrhage/siderosis	0.50	0.94	0.71	0.87	0.59	0.82	0.99
T2 hyperintensity	0.78	0.79	0.60	0.90	0.68	0.79	1
Peripheral enhancement	0.51	0.93	0.87	0.68	0.64	0.74	0.98
Washout	0.64	0.82	0.50	0.89	0.56	0.64	0.64
Mean values	0.71	0.89	0.78	0.86	0.73	0.84	-
SD values	0.17	0.08	0.14	0.09	0.13	0.10	-

PPV = positive predictive value, NPV = negative predictive value, AUC = area under the receiver operator characteristic curve, SD = standard deviation.

**Table 4:** Results for annotated features: focal nodular hyperplasia.

Radiological Features	True Positives	True Negatives	False Positives	False Negatives	f1
Delayed phase enhancement	34	0	0	2	0.97
Iso- or hyperintensity on venous phase	34	0	0	2	0.97
Early enhancement	32	1	2	1	0.96
Central scar	18	0	17	1	0.67
Washout	0	34	2	0	0
Peripheral enhancement	0	33	3	0	0
Capsule	0	34	2	0	0
T2 hyperintensity	0	32	0	4	0
Hemorrhage/siderosis	0	32	4	0	0
Hypoenhancing core	0	36	0	0	-

**Table 5:** Results for annotated features: hepatocellular carcinoma.

Radiological Features	True Positives	True Negatives	False Positives	False Negatives	f1
Capsule	13	18	1	5	0.81
Early enhancement	19	6	1	11	0.76
Hemorrhage/siderosis	12	14	6	5	0.69
Washout	16	3	2	16	0.64
Hypoenhancing core	11	12	9	5	0.61
T2 hyperintensity	3	26	6	2	0.43
Delayed phase enhancement	0	35	2	0	0
Peripheral enhancement	0	20	17	0	0
Central scar	0	34	3	0	0
Iso- or hyperintensity on venous phase	0	34	2	1	0

**Table 6:** Results for annotated features: liver metastasis.

Radiological Features	True Positives	True Negatives	False Positives	False Negatives	f1
Peripheral enhancement	27	0	0	3	0.95
Hypoenhancing core	26	0	0	4	0.93
T2 hyperintensity	18	3	1	8	0.8
Early enhancement	0	26	4	0	0
Washout	0	25	5	0	0
Central scar	0	29	1	0	0
Capsule	0	28	2	0	0
Hemorrhage/siderosis	0	28	2	0	0
Delayed phase enhancement	0	30	0	0	-
Iso- or hyperintensity on venous phase	0	30	0	0	-

**Table 7:** Results for annotated features: other lesions.

Radiological Features	True Positives	True Negatives	False Positives	False Negatives	f1
T2 hyperintensity	4	2	0	3	0.73
Hypoenhancing core	3	2	3	1	0.6
Early enhancement	1	6	1	1	0.5
Delayed phase enhancement	0	8	1	0	0
Peripheral enhancement	0	2	6	1	0
Central scar	0	7	2	0	0
Iso- or hyperintensity on venous phase	0	8	1	0	0
Washout	0	9	0	0	-
Capsule	0	9	0	0	-
Hemorrhage/siderosis	0	9	0	0	-

**Table 8:** Interobserver agreement between the three observers, measured by Cohen’s Kappa.

Radiological Features	Model vs. Expert	Model vs. Novice	Novice vs. Expert
Delayed phase enhancement	0.90	0.76	0.82
Iso- or hyperintensity on venous phase	0.88	0.73	0.77
Capsule	0.67	0.51	0.76
Early enhancement	0.62	0.59	0.82
Hypoenhancing core	0.60	0.54	0.83
T2 hyperintensity	0.52	0.52	0.81
Hemorrhage/siderosis	0.50	0.42	0.76
Central scar	0.48	0.68	0.66
Peripheral enhancement	0.45	0.36	0.79
Washout	0.41	0.53	0.59
Mean values	0.60	0.56	0.76
SD values	0.16	0.12	0.07

## Data Availability

Datasets, final model weights, and annotations are available at the following DOI: 10.6084/m9.figshare.19495013 (accessed on 31 March 2022). The software code for the experiments is available at https://github.com/stollmayer/feature_identifier (accessed on 31 March 2022). Additional anonymized data are available upon request from the corresponding author.
